# Aberrant DNA Methylation Is Associated with a Poor Outcome in Juvenile Myelomonocytic Leukemia

**DOI:** 10.1371/journal.pone.0145394

**Published:** 2015-12-31

**Authors:** Hirotoshi Sakaguchi, Hideki Muramatsu, Yusuke Okuno, Hideki Makishima, Yinyan Xu, Yoko Furukawa-Hibi, Xinan Wang, Atsushi Narita, Kenichi Yoshida, Yuichi Shiraishi, Sayoko Doisaki, Nao Yoshida, Asahito Hama, Yoshiyuki Takahashi, Kiyofumi Yamada, Satoru Miyano, Seishi Ogawa, Jaroslaw P. Maciejewski, Seiji Kojima

**Affiliations:** 1 Department of Pediatrics, Nagoya University Graduate School of Medicine, Nagoya, Japan; 2 Department of Hematology and Oncology, Children’s Medical Center, Japanese Red Cross Nagoya First Hospital, Nagoya, Japan; 3 Department of Translational Hematology and Oncology Research, Taussig Cancer Institute, Cleveland Clinic, Cleveland, Ohio, United States of America; 4 Department of Pathology and Tumor Biology, Graduate School of Medicine, Kyoto University, Kyoto, Japan; 5 Department of Neuropsychopharmacology and Hospital Pharmacy, Nagoya University Graduate School of Medicine, Nagoya, Japan; 6 Laboratory of DNA information Analysis, Human Genome Center, Institute of Medical Science, The University of Tokyo, Tokyo, Japan; Queen's University Belfast, UNITED KINGDOM

## Abstract

Juvenile myelomonocytic leukemia (JMML), an overlap of myelodysplastic / myeloproliferative neoplasm, is an intractable pediatric myeloid neoplasm. Epigenetic regulation of transcription, particularly by CpG methylation, plays an important role in tumor progression, mainly by repressing tumor-suppressor genes. To clarify the clinical importance of aberrant DNA methylation, we studied the hypermethylation status of 16 target genes in the genomes of 92 patients with JMML by bisulfite conversion and the pryosequencing technique. Among 16 candidate genes, *BMP4*, *CALCA*, *CDKN2A*, and *RARB* exhibited significant hypermethylation in 72% (67/92) of patients. Based on the number of hypermethylated genes, patients were stratified into three cohorts based on an aberrant methylation score (AMS) of 0, 1–2, or 3–4. In the AMS 0 cohort, the 5-year overall survival (OS) and transplantation-free survival (TFS) were good (69% and 76%, respectively). In the AMS 1–2 cohort, the 5-year OS was comparable to that in the AMS 0 cohort (68%), whereas TFS was poor (6%). In the AMS 3–4 cohort, 5-year OS and TFS were markedly low (8% and 0%, respectively). Epigenetic analysis provides helpful information for clinicians to select treatment strategies for patients with JMML. For patients with AMS 3–4 in whom hematopoietic stem cell transplantation does not improve the prognosis, alternative therapies, including DNA methyltransferase inhibitors and new molecular-targeting agents, should be established as treatment options.

## Introduction

Juvenile myelomonocytic leukemia (JMML) is an aggressive myeloid neoplasm of early childhood characterized by the excessive proliferation of myelomonocytic cells and hypersensitivity to granulocyte-macrophage colony-stimulating factor [1]. JMML is categorized as an overlap of myelodysplastic syndrome and myeloproliferative neoplasm by the World Health Organization classification [2]. Mutations in *PTPN11*, *NF1*, *NRAS*, *KRAS*, or *CBL*, which induce deregulation of the RAS signaling pathway, are responsible for the pathogenesis of up to 80% of cases of JMML [3–5]. Recently, we identified secondary somatic mutations in *SETBP1* and *JAK3* in patients with JMML that were associated with poor prognosis [6–8]. The clinical management of patients with JMML according to the genetic status is of great interest because of the possible prognostic variety in JMML; however, this idea remains controversial [9–10].

Methylation of cytosines in promoter CpG dinucleotides is an important negative regulator of gene expression in the human genome. Aberrant methylation of CpG islands in the promoter regions of tumor-suppressor genes plays a fundamental role in the pathogenesis of various cancers [11,12], including myeloid malignancies [13,14]. Several studies have described aberrant DNA methylation in JMML [15,16], but most researchers studied small cohorts, excluding a report of 127 cases of JMML by the European Working Group of MDS (EWOG-MDS) showing that aberrant DNA methylation of four genes (*BMP4*, *CALCA*, *CDKN2B*, and *RARB)* is associated with poor prognosis [17]. However, no report has confirmed this observation because JMML is extremely rare. In this study, to further clarify the clinical importance of aberrant DNA methylation in JMML, we quantitatively evaluated the DNA methylation pattern in the promoter regions of 16 selected genes using pyrosequencing of genomic DNA from bone marrow specimens at diagnosis from 92 patients with JMML.

## Materials and Methods

### Patients

We studied 92 children (61 males and 31 females) with JMML or Noonan syndrome-associated myeloproliferative disorder (NS-MPD) who were diagnosed in various institutions throughout Japan. The diagnosis of JMML was based on internationally accepted criteria [2]. Written informed consent was obtained from each patient’s parents before sample collection. This study was approved by the Ethics Committees of Nagoya University Graduate School of Medicine. The clinical records of patients were obtained from each institution. The detailed patient characteristics were described in our previous report^7^ and are summarized in Table 1. With genetic analysis, mutations in *PTPN11*, *NF1*, *NRAS*, *KRAS*, and *CBL* were found in 39 (42%), 7 (8%), 12 (13%), 13 (14%), and 11 (12%) patients, respectively. In addition, 16 patients had *SETBP1* or *JAK3* mutations [7].

**Table 1:** Patient characteristics.

	Total cohort (n = 92)	AMS 0 (n = 25)	AMS 1–2 (n = 54)	AMS 3–4 (n = 13)
Gender (Male/Female)	61/31	13/12	39/15	9/4
Median age at diagnosis, months (range)	16 (1–160)	8 (1–59)	23 (1–160)	29 (1–74)
Diagnosis (JMML/NS-MPD)	85/7	20/5	52/2	13/0
Hypermethylated genes, n				
*BMP4*	41	0	29	12
*CALCA*	32	0	20	12
*CDKN2A*	32	0	20	12
*RARB*	13	0	9	4
Genetic mutations in the RAS pathway, n				
*PTPN11*	39	6	26	7
*NF1*	9	1	5	3
*RAS (NRAS/KRAS)*	28 (15/13)	8 (5/3)	18 (9/9)	2 (1/1)
*CBL*	14	7	3	3
No mutation	10	4	5	1
Secondary genetic mutations, n				
*SETBP1* and *JAK3*	1	0	1	0
*SETBP1* only	6	0	5	1
*JAK3* only	9	0	5	4
No secondary mutation	76	25	43	8
Metaphase cytogenetics at diagnosis, n				
Normal karyotype	76	24	41	11
Monosomy 7	8	1	7	0
Trisomy 8	4	0	3	1
Other abnormalities	4	0	3	1
WBC at diagnosis, ×10^9^/L, median (range)	30.0 (1.0–563)	26.9 (1.0–131)	29.7 (5.6–113)	43.1 (15.3–563)
Mon at diagnosis, ×10^9^/L, median (range)	4.6 (0.2–31.6)	4.5 (0.2–18.4)	4.5 (0.5–20.8)	5.8 (1.1–31.6)
HbF at diagnosis, %, median (range)	21 (0–68)	8 (0–68)	22 (0–63)	24 (8–56)
PLT at diagnosis, ×10^9^/L, median (range)	61.0 (1.4–483)	112 (5.0–373)	53.0 (1.4–483)	49.0 (8–132)
HSCT (Yes/No)	56/36	6/19	43/11	7/6
Probability of 5-year TFS, % (95% CI)	15 (6–27)	69 (45–84)	6 (1–17)	0
Survival outcome (Alive/Dead)	62/30	20/5	41/13	1/12
Probability of 5-year OS, % (95% CI)	60 (46–71)	76 (45–91)	68 (50–81)	8 (0–29)

Abbreviations: AMS, aberrant methylation score; CI, confidential interval; HbF, hemoglobin F; HSCT, hematopoietic stem cell transplantation; JMML, juvenile myelomonocytic leukemia; Mon, monocytes; NS-MPD, Noonan syndrome-associated myeloproliferative disorder; OS, overall survival; PLT, platelet count; TFS, transplantation-free survival; WBC, white blood cell count.

### Laboratory methods

We examined genomic DNA derived from bone marrow specimens at the diagnosis of JMML or NS-MPD. As normal controls, age- and tissue-matched samples were obtained from 16 healthy donors. Genomic DNA was extracted from the peripheral blood of normal controls and bone marrow specimens of children with JMML using a QIAamp DNA Blood Mini kit (QIAGEN, Hilden, Germany) and a QIAamp DNA Investigator kit (QIAGEN), according to the manufacturer’s instructions. The DNA was bisulfite-modified using an EpiTect Bisulfite kit (QIAGEN). We quantified CpG methylation in the promoter regions of 16 candidate genes (*APC*, *BMP4*, *CALCA*, *CDH13*, *CDKN2A*, *CDKN2B*, *CHFR*, *DAPK1*, *ESR1*, *H19*, *IGF2AS*, *MGMT*, *MLH1*, *RARB*, *RASSF1*, and *TP73*), which were reported to be highly methylated in JMML [15–17], or other hematological malignancies [18–25]. Bisulfite-converted DNA was amplified using a PyroMark PCR kit (QIAGEN) with specific primers for the CpG islands of target genes [26]. The informations of the region of each target genes investigated were listed in S1 File. To calculate the DNA methylation level of each targeted CpG, the peak heights in the resulting pyrogram describe the ratio of cytosine to thymine at each analyzed CpG site, which reflects the proportion of methylated DNA, and the average methylation of the all CpG sites for each gene was calculated [27]. We defined aberrant hypermethylation as higher than three standard deviations above the mean of the methylation level in normal controls.

### Statistical analyses

Comparisons of proportions were performed using the χ^2^ test for heterogeneity or Fisher’s exact test as appropriate. Mean ranks were compared with the Wilcoxon two-sample test. All candidate predictive factors were investigated for their impact on the duration of transplantation-free survival (TFS), which was defined as the interval from diagnosis to hematopoietic stem cell transplantation (HSCT) or death of any cause, and overall survival (OS), which was defined as the time from diagnosis to death regardless of disease status using the Kaplan–Meier estimate. The observation time of patients who had not progressed and who were alive at the last follow-up visit was considered to be censored. Covariates with a *p*-value <0.10 in univariate analyses were included in multivariate analyses. As estimates of the prognostic effect, hazard ratios (HR) with 95% confidential intervals (CI) were calculated with Cox proportional hazard models. All reported *p*-values were two-sided and the significance level was set at 0.05. Statistical analyses were performed using Stata 12.0 (Stata Corporation, College Station, TX, USA).

## Results

**Fig 1:**
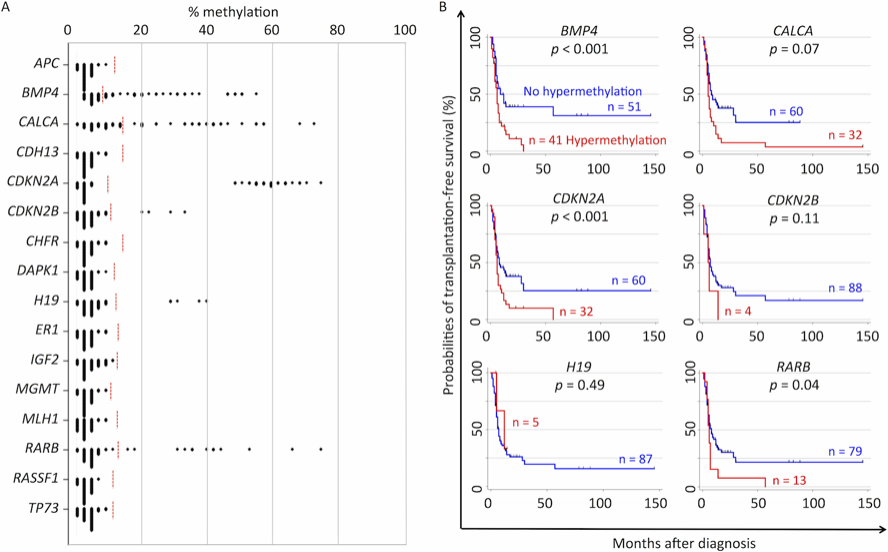
Summary of DNA methylation in candidate genes. (A) The dot plot represents the frequencies of methylated CpG sites for each candidate gene in the 92 patients with juvenile myelomonocytic leukemia. Aberrant hypermethylation was defined as >3 standard deviations above the mean methylation level of the healthy control population. The threshold values of each gene are shown as red broken lines. (B) Kaplan–Meier plots of the patient groups, defined by aberrant methylation of the indicated genes, are shown for *BMP4*, *CALCA*, *CDKN2A*, *CDKN2B*, *H19*, and *RARB*.

Among 16 candidate genes, aberrant hypermethylation of *BMP4* (41/92, 45%), *CALCA* (32/92, 35%), *CDKN2A* (32/92, 35%), *CDKN2B* (4/92, 4%), *H19* (5/92, 5%), and *RARB* (13/92, 14%) was detected in our cohort (Fig 1A). For the other 10 genes, no hypermethylation exceeding the limit of detection was observed. Hypermethylation of *BMP4*, *CALCA*, *CDKN2A*, and *RARB* in particular, resulted in a poorer outcome as indicated by the Kaplan–Meier estimates for TFS, for which the probabilities of TFS for patients with and without methylation were: 31% and 0% (*p* <0.001), for *BMP4*; 25% and 4% (*p* = 0.07), for *CALCA*; 25% and 0% (*p* <0.001), for *CDKN2A*; and 22% and 0% (*p* = 0.04), for *RARB*, respectively (Fig 1B).

**Fig 2:**
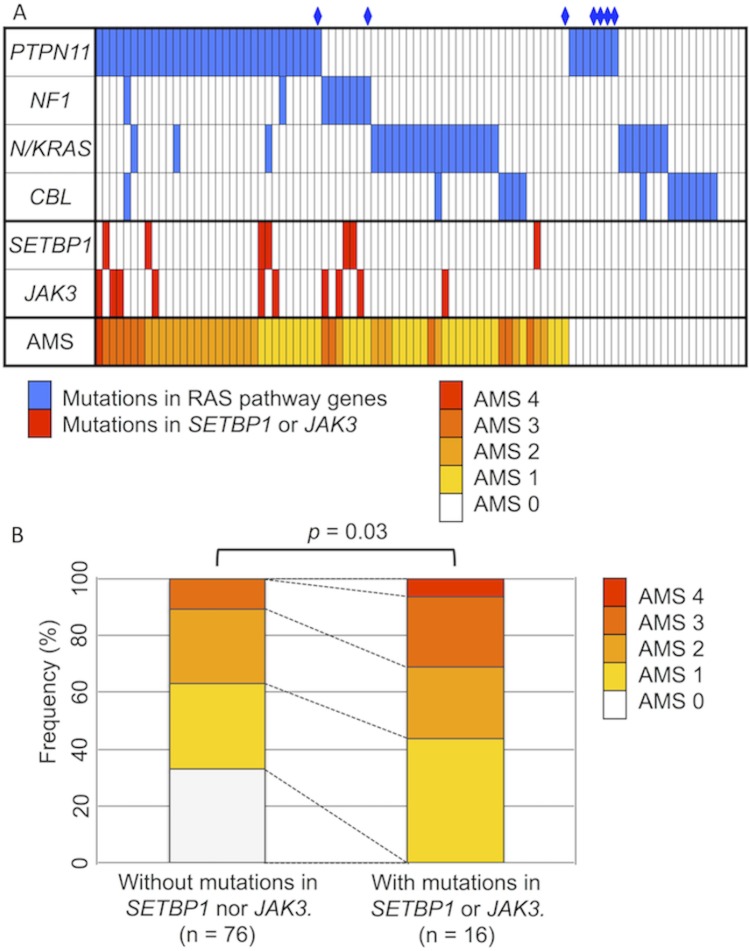
Profile of genetic mutations and aberrant methylation. (A) Mutation status of RAS pathway genes and secondary genes (*SETBP1* and *JAK3*) identified as gene targets. Aberrant methylation scores (AMS) in a cohort of 92 patients with juvenile myelomonocytic leukemia are summarized. A rhombus denotes a patient with Noonan syndrome-associated myeloproliferative disorder. (B) Mutations in *SETBP1* and *JAK3* were associated with a higher AMS. The mean AMS of patients with *SETBP1* and/or *JAK3* mutations was higher than that of patients without secondary mutations (p = 0.03).

Thus, we considered that four of the candidate genes were aberrantly hypermethylated (*BMP4*, *CALCA*, *CDKN2B*, and *RARB*) and assigned an aberrant methylation score (AMS; one point for each methylated gene for these four genes) to further assess the genetic and clinical impact of the hypermethylation. Accordingly, 25 (27%), 30 (33%), 24 (26%), 12 (13%), and one (1%) patient had AMSs of 0, 1, 2, 3, and 4, respectively. Among the 7 patients with NS-MPD, three and four had AMSs of 0 and 1, respectively, however, none of the patients had AMS of 2 or more. The AMSs in the cohort of 92 patients with JMML are summarized in Fig 2A. Of the 16 patients with mutations in *SETBP1* and/or *JAK3*, 0 (0%), 7 (44%), 4 (25%), 4 (25%), and one (6%) patient had AMSs of 0, 1, 2, 3, and 4, respectively. However, of the other 76 patients without mutations in *SETBP1* or *JAK3*, 25 (33%), 23 (30%), 20 (26%), 8 (11%), and 0 (0%) patients had AMSs of 0, 1, 2, 3, and 4, respectively. As shown in Fig 2B, the AMS of patients with *SETBP1* and/or *JAK3* mutations was significantly higher than that in patients without these secondary mutations (*p* = 0.03).

**Fig 3:**
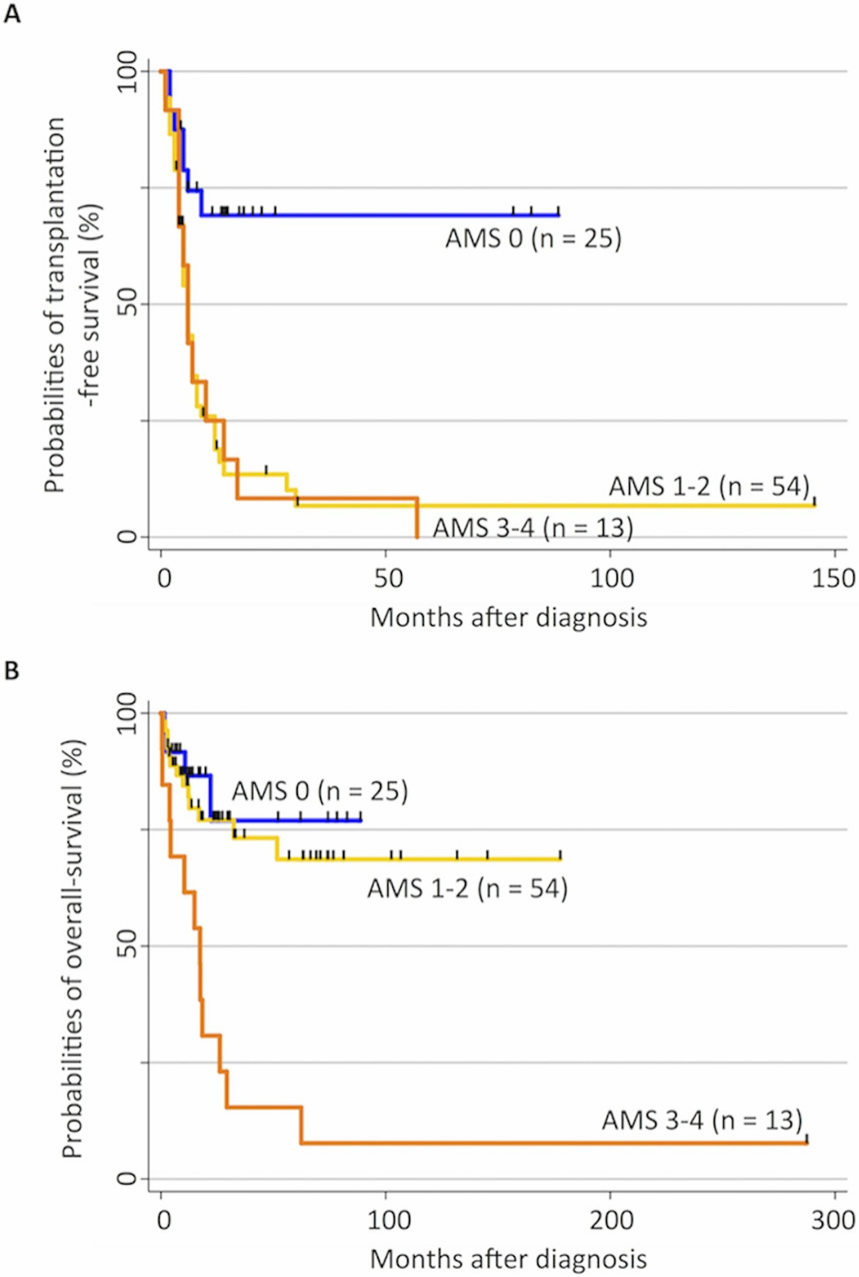
Hypermethylation status and clinical outcome in patients with juvenile myelomonocytic leukemia (JMML). (A) Kaplan–Meier curves represent the probability of transplantation-free survival (TFS) in the 92 patients with JMML. TFS was defined as the probability of being alive and transplantation free. Both death and transplantation were considered events. The probability of 5-year TFS in the aberrant methylation score (AMS) 0 cohort (solid line) was significantly higher than that in the AMS 1–2 (long dashed line) and AMS 3–4 cohorts (dashed line), p < 0.001. (B) Kaplan–Meier curves represent the probability of overall survival (OS) in the 92 patients with JMML. Death was considered an event. The probability of OS in both the AMS 0 (solid line) and 1–2 cohorts (long dashed line) was significantly higher than that in the AMS 3–4 cohort (dashed line), p < 0.001.

The patients were classified into three cohorts according to the AMS score (Table 1 and Fig 3). The AMS 0 cohort displayed better survival without HSCT; specifically, the probabilities of 5-year TFS and OS in this cohort were 69% (95% CI, 45%–84%) and 76% (95% CI, 45%–91%), respectively. Patients in the AMS 1–2 cohort were rescued by HSCT. The probabilities of 5-year TFS and OS in this cohort were 6% (95% CI, 1%–17%) and 68% (95% CI, 50%–81%), respectively. In sharp contrast, the prognosis of patients in the AMS 3–4 cohort was not improved by HSCT, as the probabilities of 5-year TFS and OS in this cohort were 0% and 8% (95% CI, 0%–29%), respectively. Indeed, six of seven patients who received allogeneic HSCT in the AMS 3–4 cohort died during observation. Their causes of death consisted of progressive disease (n = 3), veno-occlusive disease (n = 2), and respiratory distress (n = 1), as shown in Table 2.

**Table 2:** Patient characteristics of the AMS 3–4 cohort.

UPN	Age (months) / Gender	AMS	Mutation in RAS pathway genes	Mutation in *JAK3* or *SETBP1*	Metaphase cytogenetics	First HSCT	Second HSCT	Observation period from diagnosis (months)	Outcome	Cause of death
2	67 / M	3	*PTPN11*	−	46,XY	−	−	4	Dead	Disease progression
4	3 / F	3	*CBL*	−	46,XX	MSD BMT	−	57	Alive	-
6	74 / F	3	*NF1*	-	46,XX	−	−	10	Dead	Disease progression
7	52 / M	3	*PTPN11*	-	46,XY	−	−	17	Dead	Disease progression
17	41 / M	3	*PTPN11*	*SETBP1*	47,XY,+8	UR CBT	−	6	Dead	Disease progression
19	1 / F	3	-	-	46,XX	−	−	4	Dead	Disease progression
21	29 / M	3	*PTPN11*	*JAK3*	45,XY, del(6)(q?),-20	UR CBT	−	4	Dead	VOD
41	3 / M	3	*PTPN11*	-	46,XY	−	−	1	Dead	Respiratory distress
43	11 / F	3	*CBL*	-	46,XX	−	−	0	Dead	Pulmonary bleeding
45	43 / M	4	*PTPN11*	*JAK3*	46,XY	UR BMT	UR CBT	6	Dead	VOD
48	23 / M	3	*NF1*	*JAK3*	46,XY	MMRD BMT	−	5	Dead	Disease progression
49	65 / M	3	*PTPN11*	*JAK3*	46,XY	UR CBT	UR BMT	14	Dead	Disease progression
53	9 / M	3	*KRAS*	-	46,XY	UR BMT	−	7	Dead	Respiratory distress

Abbreviations: AMS, aberrant methylation score; BMT, bone marrow transplantation; CBT, cord blood transplantation; F, female; HSCT, hematopoietic stem cell transplantation; M, male; MMRD, mismatched related donor; MSD, matched sibling donor; UR, unrelated; VOD, veno-occlusive disease.

According to multivariate analysis using the Cox proportional hazard model for covariates including AMS (Table 3), an AMS of 1–4 (HR, 4.1; 95% CI, 1.5–11; *p* = 0.006), mutations in *PTPN11* or *NF1* (HR, 3.5; 95% CI, 1.6–7.5; *p* = 0.002), and chromosomal aberrations (HR, 2.3; 95% CI, 1.1–4.7; *p* = 0.021) were independent predictors of poor TFS. Moreover, an AMS of 3–4 (HR, 4.4; 95% CI, 1.7–11; *p* = 0.002), chromosomal aberrations (HR, 4.1; 95% CI, 1.6–11; *p* = 0.004), and platelet counts of less than 33 × 10^9^/L (HR, 3.1; 95% CI, 1.4–7.3; *p* = 0.007) were independent predictors of poor OS. On the contrary, in multivariate analysis for covariates excluding AMS (Table 3), mutations in *PTPN11* or *NF1* (HR, 2.1; 95% CI, 1.2–5.1; *p* = 0.01), chromosomal aberrations (HR, 3.7; 95% CI, 1.9–7.1; *p* <0.001), and platelet counts of less than 33 x 10^9^/L (HR, 2.0; 95% CI, 1.1–3.5; *p* = 0.02) were independent predictors of poor TFS. Furthermore, chromosomal aberrations (HR, 3.8; 95% CI, 1.5–9.8; *p* = 0.006) and platelet counts of less than 33 × 10^9^/L (HR, 2.8; 95% CI, 1.2–6.2; *p* = 0.015) were independent predictors of poor OS. Comparing these analyses, the AMS refined the predictability of outcomes (S1 Fig).

**Table 3:** Multivariate model for transplantation-free and overall survival.

Covariates	Model without aberrant methylation score				Model with aberrant methylation score			
	Transplantation-free survival		Overall survival		Transplantation-free survival		Overall survival	
	HR (95% CI)	p-value	HR (95% CI)	p-value	HR (95% CI)	p-value	HR (95% CI)	p-value
Mutation in *PTPN11* or *NF1*	2.5 (1.2–5.1)	0.01	2.1 (0.6–7.1)	0.24	3.5 (1.6–7.5)	0.002	2.1 (0.6–7.7)	0.24
Chromosomal aberration	3.7 (1.–7.1)	<0.001	3.8 (1.5–9.8)	0.006	2.3 (1.1–4.7)	0.021	4.1 (1.6–11)	0.004
Age at diagnosis >48 months	1.0 (0.5–1.8)	0.94	2.2 (0.9–5.2)	0.09	0.7 (0.4–1.4)	0.35	1.8 (0.7–4.5)	0.2
HbF >15%	1.7 (0.8–3.7)	0.15	1.9 (0.5–7.4)	0.33	1.0 (0.4–2.2)	0.92	1.2 (0.3–5.2)	0.79
PLT <33 × 10^9^/L	2.0 (1.1–3.5)	0.02	2.8 (1.2–6.2)	0.015	1.6 (0.9–3.0)	0.1	3.1 (1.4–7.3)	0.007
Aberrant methylation score ≥1	–	–	–	–	4.1 (1.5–11)	0.006	4.4 (1.7–11)	0.002

Abbreviations: CI, confidence interval; HbF, hemoglobin F; HR, hazard ratio; PLT, platelets.

## Discussion

Negative regulation by aberrant methylation of the CpG islands of tumor-suppressor genes accounts for the pathogenesis of malignancy [11,12]. In this study, we analyzed JMML cells from 92 children to measure the CpG methylation of 16 candidate genes by bisulfite conversion and pyrosequencing techniques. Aberrant methylation of six genes (*BMP4*, *CALCA*, *CDKN2A*, *CDKN2B*, *H19*, and *RARB*) was detected in 4%–45% of our cohort. Four of these genes (*BMP4*, *CALCA*, *CDKN2B*, and *RARB*) were consistent with those identified in the EWOG-MDS report, which described that CpG hypermethylation of these four genes was significantly associated with poor prognosis in JMML. Of note, the method for measuring methylation in the current study was different from that used in the EWOG-MDS study [17], suggesting that both methods can analyze targeted CpG methylation, supporting the reasonability of both studies.

In our study, aberrant methylation of four genes (*BMP4*, *CALCA*, *CDKN2A*, and *RARB*) was associated with poor prognosis. In contrast, EWOG-MDS study incorporated *CDKN2B* in the methylated gene sets, while *CDKN2A* was not included. In the current study, patients with an aberrant methylation of *CDKN2B* tended to show a poorer outcome (p = 0.11); however, in the current cohort, the size of the population with aberrant *CDKN2B* was small (4 of 92) compared with the population size (28 of 127) observed in the EWOG study. This difference may reflect the use of different methodologies in the two studies (pyrosequencing in the present study and MassARRAY in the EWOG study). *CDKN2A*, also known as *p16*, is coded in 9p21, and binds to *CDK4*, inhibiting the ability of *CDK4* to interact with cyclin D and stimulate passage through the G1 phase of the cell cycle [28]. The role of *CDKN2A* in leukemogenesis is well investigated. The first report in 1994 demonstrated that both alleles of *p16* were completely or partially deleted in human leukemia cells derived from patients [29]. Hemizygous deletions and rearrangements of 9p21 are reported as among the most frequent cytogenetic abnormalities detected in pediatric acute lymphoblastic leukemia [30]. A genome-wide analysis demonstrated that a common variation at 9p21.3 (intron 1 of *CDKN2A*) influences the risk of childhood acute lymphoblastic leukemia [31].

The current study indicated that aberrant methylation of multiple genes is a common phenomenon in JMML; specifically, 73% of patients exhibited methylation of at least one of four genes (*BMP4*, *CALCA*, *CDKN2A*, and *RARB*), whereas 40% of all patients had two or more genes methylated. These observations suggest that the patient cohort of JMML includes a CpG island methylator phenotype [17,32]. Moreover, methylation of multiple genes was significantly associated with mutations in *SETBP1* or *JAK3*, which were involved in the progression rather than initiation of JMML [7]. Both aberrant methylation and secondary genetic mutations are associated with poor clinical outcomes, reflecting the aggressive disease at diagnosis of JMML. Indeed, our analyses identified that aberrant methylation is one of the independent poor prognosis factors for outcomes and refined the predictability of outcomes. The prognosis of patients with hypermethylation in three or all four genes was not improved by HSCT, as the most frequent cause of death in these patients was progression of JMML despite HSCT.

The most exciting finding in the current study was that patients with JMML could be classified using the AMS. The AMS 0 cohort exhibited significantly better TFS than the AMS 1–2 and 3–4 cohorts. At the same time, the AMS 0 and 1–2 cohorts displayed significantly better OS than the AMS 3–4 cohort. These results suggest that allogeneic HSCT improves the survival of patients with an AMS of 1–2 but not 3–4, providing new insight into clinical decision-making for patients with JMML. First, patients without aberrant methylation may survive without allogeneic HSCT, indicating that for careful observation, a more specific prognostic marker may need to be identified. Second, for patients with an AMS of 1–2, we provided evidence that early HSCT may improve their outcomes. Finally, for patients with an AMS of 3–4 for whom HSCT does not improve the prognosis, alternative therapies, including DNA methyltransferase inhibitors and new molecular-targeting agents, should be established as treatment options.

Epigenetic analysis provides helpful information for clinicians to select treatment strategies for patients with JMML. Our findings unmasked a new aspect of the molecular pathogenesis of JMML, which could facilitate the development of novel therapies; however, the study had some limitations. We assessed methylation using materials obtained only at diagnosis but not at later time points. In addition, our assessments were done for only 16 selected genes, whereas genome-wide profiling was not performed. Moreover, our analyses were possibly influenced by race bias. As JMML is an extremely rare disease, an international prospective study with risk classification by genetic and epigenetic analysis is warranted.

## Supporting Information

S1 Fig Kaplan–Meier curves for transplantation-free and overall survival stratified by risk groups based on a multivariate survival model. Patients were divided into three groups based on a multivariate analysis model lacking the aberrant methylation score (AMS), i.e., the presence of a PTPN11 or NF1 mutation, chromosomal aberration, and low platelet count (<33 × 109/L). We defined patients as having low (Low; without any covariates), intermediate (Int; with 1 covariate), and high risk (High; with ≥2 covariates). When we incorporated AMS in the patient stratification, patients were divided into three groups based on a multivariate analysis model including AMS (AMS model), i.e., the presence of a PTPN11 or NF1 mutation, chromosomal aberration, low platelet count (<33 × 109/L), and high AMS (AMS ≥1 for TFS). We defined patients as having low (Low-M; without any covariates), intermediate (Int-M; with 1 covariates), and high risk (High-M; with ≥3 covariates) using the AMS model. Kaplan–Meier curves represent the probabilities of TFS based on the two models (panels A, B).
(TIFF)

S1 File Summary of targeted CpGs in candidate genes. Primers for targeted CpGs in each gene, excluding RARB, were supplied by commercial companies. Thus, the primers for APC (product code #ASY516), BMP4 (#ADS1490), CALCA (#ADS1489), CDH13 (#ADS1461), CDKN2B (#ADS708), CHFR (#ADS1462), DAPK1 (#ADS040), ESR1 (#ADS076), H19 (#ADS596), IGF2 (#ADS1051), and RASSF1 (#ASY574) were supplied by EPIGENDX (Hopkinton, MA, USA), while those for TP73 (#ADS658), CDKN2A (#972012), MGMT (#972032), and MLH1 (#972022) were supplied by QIAGEN. PCR primers and the sequencing primer for the CpGs of RARB were as reported by Shaw et al. The sequence informations of targeted CpGs in candidate genes were summarized as bellow.
(PDF)
