# Supplementary figures and images for: Aberrant DNA Methylation Is Associated with a Poor Outcome in Juvenile Myelomonocytic Leukemia
Source: PLoS One. 2015 Dec 31;10(12):e0145394. doi: 10.1371/journal.pone.0145394 (PMC4697810; doi:10.1371/journal.pone.0145394)

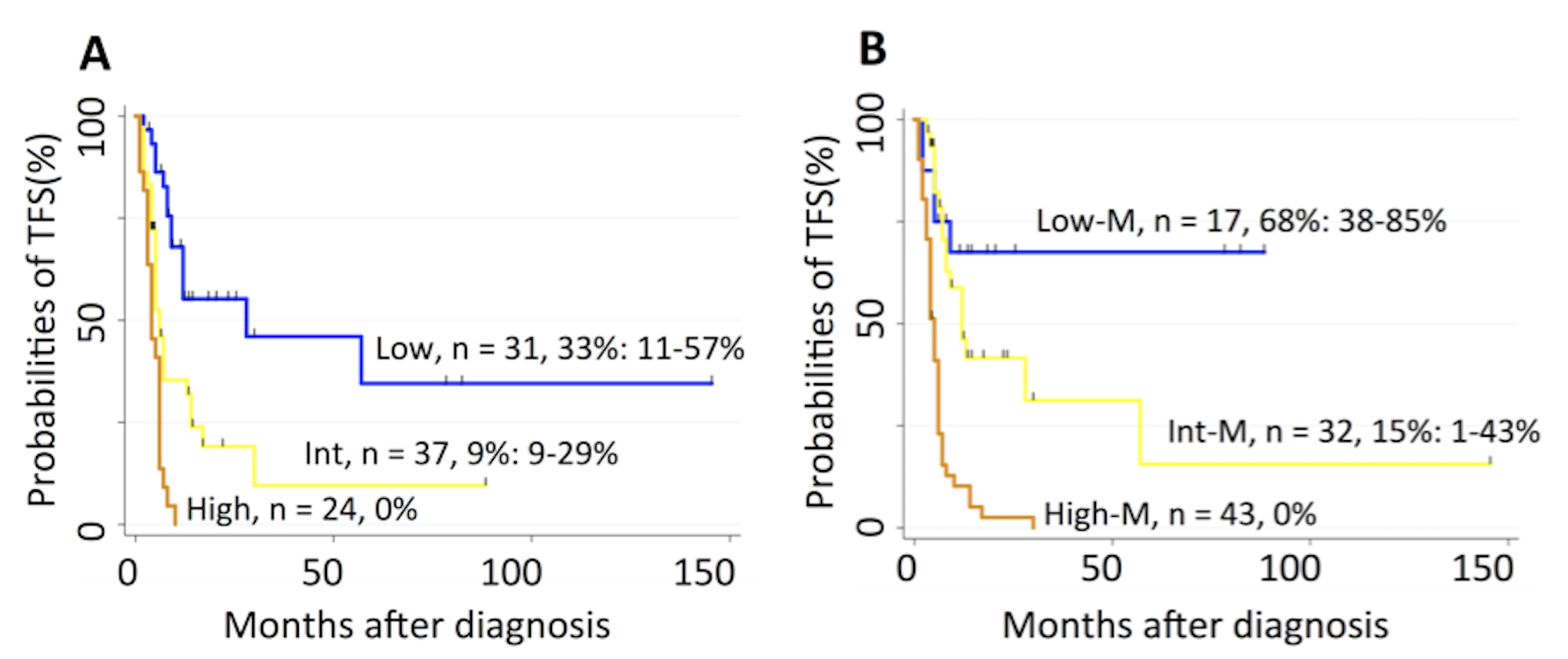

Supplement: S1 Fig — Patients were divided into three groups based on a multivariate analysis model lacking the aberrant methylation score (AMS), i.e., the presence of a PTPN11 or NF1 mutation, chromosomal aberration, and low platelet count (<33 × 109/L). We defined patients as having low (Low; without any covariates), intermediate (Int; with 1 covariate), and high risk (High; with ≥2 covariates). When we incorporated AMS in the patient stratification, patients were divided into three groups based on a multivariate analysis model including AMS (AMS model), i.e., the presence of a PTPN11 or NF1 mutation, chromosomal aberration, low platelet count (<33 × 109/L), and high AMS (AMS ≥1 for TFS). We defined patients as having low (Low-M; without any covariates), intermediate (Int-M; with 1 covariates), and high risk (High-M; with ≥3 covariates) using the AMS model. Kaplan–Meier curves represent the probabilities of TFS based on the two models (panels A, B). (TIFF) [file pone.0145394.s001.tiff]
