# Supplementary material for: Aberrant DNA Methylation Is Associated with a Poor Outcome in Juvenile Myelomonocytic Leukemia
Source: PLoS One. 2015 Dec 31;10(12):e0145394. doi: 10.1371/journal.pone.0145394 (PMC4697810; doi:10.1371/journal.pone.0145394)
Supplement: S1 File — Primers for targeted CpGs in each gene, excluding RARB, were supplied by commercial companies. Thus, the primers for APC (product code #ASY516), BMP4 (#ADS1490), CALCA (#ADS1489), CDH13 (#ADS1461), CDKN2B (#ADS708), CHFR (#ADS1462), DAPK1 (#ADS040), ESR1 (#ADS076), H19 (#ADS596), IGF2 (#ADS1051), and RASSF1 (#ASY574) were supplied by EPIGENDX (Hopkinton, MA, USA), while those for TP73 (#ADS658), CDKN2A (#972012), MGMT (#972032), and MLH1 (#972022) were supplied by QIAGEN. PCR primers and the sequencing primer for the CpGs of RARB were as reported by Shaw et al. The sequence informations of targeted CpGs in candidate genes were summarized as bellow. (PDF) [file pone.0145394.s002.pdf]

Table SI. Summary of targeted CpGs in candidate genes.

Primers for targeted CpGs in each gene, excluding RARB, were supplied by commercial companies. Thus, the primers for APC (product code #ASY516), BMP4 (#ADS1490), CALCA (#ADS1489), CDH13 (#ADS1461), CDKN2B (#ADS708), CHFR (#ADS1462), DAPK1 (#ADS040), ESR1 (#ADS076), H19 (#ADS596), IGF2 (#ADS1051), and RASSF1 (#ASY574) were supplied by EPIGENDX (Hopkinton, MA, USA), while those for TP73 (#ADS658), CDKN2A (#972012), MGMT (#972032), and MLH1 (#972022) were supplied by QIAGEN. PCR primers and the sequencing primer for the CpGs of RARB were as reported by Shaw et al. The sequence informations of targeted CpGs in candidate genes were summarized as bellow.

Green highlighted G is the transcriptional start site.
Yellow highlighted ATG is the translational start site.
Gray highlighted CGs are covered and assessed.

APC

ENSG00000134982

-187 to -47 from transcription starting site (TSS)

**cggttgggcggggccctgtgccccactgcggagtgcgggtcgggaagcggagagagaagcagctgtgtaatcc
gctggatgcggaccagggcgctccccattcccgtcgggagcccgccgattggctgggtgtgggcgcacgtgacc
gacatgtggctgtattggtgcagcccgccagggtgtcactGGAGACAGAATGGAGGTGCTGCCGGA
CTCGGAAATGGG**

BMP4

ENSG00000125378

-317 to -152 from TSS

**cggcgactagtcggtgcatgctttctagtacctccgcacgtggtccccaggtgagccccagccgcttcccagagct
ggaggcagcggcgtcccagctccgacggcagctgcggactcgggcgctgcctgggcttccgggacccgggcc
tgctaggcgaggtcgggcggctggaggggaggatgtgggcggggctcccatccccagaaagggaggcgagc
gagggaggagggaaggagggaggggccgccggggaagaggaggaggaaggaaagaaagaaagcgaggg**

agggaaagaggaggaaggaagatgcgagaaGGCAGAGGAGGAGGGAGGGA

CALCA

ENSG00000110680

-331 to -78 from TSS

cgccaaatcgtgaaaccaggggtggagtggggcgagggttcaaaaccaggccggactgagaggtgaaattcac catgacgtcaaactgccctcaaattcccgctcactttaagggcgttacttgttggtgcccccaccatcccccaccattt ccatcaatgacctcaatgcaaatacaagtgggacggtcctgctggatcctccaggttctggaagcatgagggtgac gcaacccaggggcaaaggacccctccgcccattggttgctgtgcactggcggaactttcccgacccacagcggc gggaataagagcagtcgctggcgctgggaGGCATCAGAGACACTGC

CDH13

ENSG00000140945;

-266 to -144 from start codon

CGTGATTTGTGAGGCTGAGCCCCAACAGTCCAAAGAAGCAAATGGGATGCCACCT CCGCGGGGCTCGCTCCTCGCGAGGTGCTCACCCCGTATCTGCCATGCAAAACGAG GGAGCGTTAGGAAGGAATCCGTCTTGTAAAGCCATTGGTCCTGGTCATCAGCCTCT ACCCAATGCTTTCGTGATGCTGCTGCTGATCTATTTGGGAAGTTGGCTGGCTGGCG AGGCAGAGCCTCTCCTCAAAGCCTGGCTCCCACGGAAAATATGCTCAGTGCAGCC GCGTGCATGAATGAAAACGCCGCCGGGCGCTTCTAGTCGGACAAAATGCAGCCGA GAACTCCGCT

CDKN2A

ENSG00000147889

-64 to -39 from start codon

CGGACCGCGTGCGCTCGGCGGCTGCGGAGAGGGGGAGAGCAGGCAGCGGGCGG CGGGGAGCAGCATGGAGCCGGC

CDKN2B

ENSG00000147883

-318 to -141 from start codon

**CGAGGCGGGGCAGTGAGGACTCCGCGACGCGTCCGCACCCTGCGGCCAGAGCGG
CTTTGAGCTCGGCTGCGTCCGCGCTAGGCGCTTTTTCCCAGAAGCAATCCAGGCGC
GCCCGCTGGTTCTTGAGCGCCAGGAAAAGCCCGGAGCTAACGACCGGCCGCTCGG
CCACTGCACGGGGCCCCAAGCCGCAGAAGGACGACGGGAGGGTAATGAAGCTGA
GCCCAGGTCTCCTAGGAAGGAGAGAGTGCGCCGGAGCAGCGTGGGAAAGAAGGG
AAGAGTGTCGTTAAGTTTACGGCCAACGGTGGATTATCCGGGCCGCTGCGCGTCTG
GGGGCTGCGGAATGCGCGAGGA**

Green highlighted ATG is the translational start site.

Gray highlighted CGs are covered and assessed.

CHFR

ENSG00000072609

-628 to -438 from start codon

**cgaaagggtccccacgtgcactacctcgtgacaactaagatggaccgtcttctactcctaggagtcatccccagatt
agcgggctctcagaatccttggggcttccaatccggaagttccccgtcccttctgccccaacataacatggcgcc
gaccgcagccacttccgtgatccgcaggcgacgaagtcgcctggtcaggatcaaagatggccgagcgccctcca
cgcgcctactctcgcgggccactcccggctccgctttagctgccgctcttggtgagcgccgccgccagccaatcg
cggggcgcagaggcctctggccccgccccgcggatcagctgagggagccgcaatgtCTCTTGACAGCG
GCGGCGGCGCAGCCGGTTCCGGGTTCGGCGCGGGGCGGGGgtaagcgcggctggcgag
ggggctgcggcgggcggcgaagggggacctggttgcggccgtgggagggggcgcccgccggcgtccaggc
cgggctgcggggctgcgtgggcgcgggcggccgggccggggcccgcggggtggggcgggcgccgagtctc
ggcctctggggtcgggagtctccatgggcgtttccggggtctccgcagATGTGAATCCCGATGGAGCG
GCCCGA**

DAPK1

ENSG00000196730

-602 to -451 from start codon

CGCTCGGGGAAGGGGAGAGGGTGGCCACGGTGTTAGGAGAGGCGCGGGAGCCGA
GAGGTGGCGCGGGGGTGCCACCGTTGCCGCAGGCTGGAGAGAGATTGCTCCCAGT

GAGGCGCGTACCGTCTGGGCGAGGGCTTCATTCTTCCGCGGCGTCCCTGGAGGTGG
GAAAGCTGGGTGGGCATGTGTGCAGAGAAAGGGGAGGCGGGGAGGCCAGTCACTT
CCGGAGCCGGTTCTGATCCCAACAGACCGCCCAGCGTTTGGGGACGCCGACCTCGG
GGTGCCGTGGTGCCCGGCCCCACGCGCGCGCGGGGCTGAGGGGTCGGGGGCGTC
CCTGGCCGCCCAGCTTTAACAAAGGGTGCTCCTCTCCACCCCGCGAGGAGGGGCAG
CTCCGGAGACCCGGTCTTCAGCGAGCGGGGTCTTAGCGCCGGGGAGGTCTACTTCC
TTTTGGGGTTGCCATTTTACTATTATTATTGCCTTTTTTTTTTCTTCAAAAG**GACTGGAG**
ACTGATGCATGAGGGGGCTACGGAGGCGCAGGAGCGGTGGTGATGGTCTGGGAA
GCGGAGCTGAAGTGCCCTGGGCTTTGGTGAGGCGTGACAGTTTATCATGACCGTGT
TCAGGCAGGAAAACGTGGATGATTACTA

ESR1

ENSG00000091831

+62 to +119 from start codon

ATGACCATGACCCTCCACACCAAAGCATCTGGGATGGCCCTACTGCATCAGATCCA
AGGGAACGAGCTGGAGCCCCTGAACCGTCCGCAGCTCAAGATCCCCCTGGAGCGG
CCCCTGGGCGAGGTGTACCTGGA

H19

ENSG00000130600

-1997 ~ -1940 from TSS

CGCACCAGATCTTCAGGTCGGGCATTATCCACAGCCCCGTGGCCCCGGGTCACACT
CCGAGGGCTTCAGTGTCATGGCCTGGGACTCAAGTCACGCCTACTTATGTGATGAT
CACAGTGTGTTCCACCAAAATCTTACATTTTCCACATCTATCCCAGAGCACAGCTCC
GACTCCGTCTAAGGACAGCCCCCAAATCCCCAGCCTTTTACTGAACTGACAATTGC
CTCCCCAGTGAACACTCTGATCTCCTCAGCCCTAAGTGGCCAGACATTAACATTCTC
ATTCAATGCAGGTTTGAGGTGCTAATTCAGGAGCTTAA……gccctgctctgattggccggc
agggcaggggcgggaattctgggcggggccaccccagttagaaaagcccgggctaggaccgaggagcAG

IGF2AS

ENSG00000099869

AY375532

nt 8605-8874

CGAATGGCCCGCCTTGAGGGGTCATGGCACGGAATATGAAAGCCTCCTCCACCTC
CAAACACCCCCACCTTGGAA[G/A]GAGATAAGGAGGGGGCCCCAGCAAAAGCCAC
TGGACACACAGCTCTGCTTGACGAGGCCAGTGAGGGACGGCGTGGCTGTGCTTCC
TGGGGAA[A/G]TGAACCCCCTCCCCACAGACACCACCCTGGGTGGAT[T/C]GAGGA
GTCTGGG

MGMT

ENSG00000170430

+28 to +47 from start codon

ATGCTGGGACAGCCCGCGCCCCTAGAACGCTTTGCGTCCCGACGCCCGCAGGT

MLH1

ENSG00000076242

-185 to -162 from TSS

CGCCAAGCACCTCCTCCGCTCTGCGCCAGATCACCTCAGCAGAGGCACACAAGCCC
GGTTCCGGCATCTCTGCTCCTATTGGCTGGATATTTCGTATTCCCCGAGCTCCTAAAA
ACGAACCAATAGGAAGAGCGGACAGCGATCTCTAACGCGCAAGCGCATATCCTTCTA
GGTAGCGGGCAGTA**GCCGCTTCAGGGAGGGAC**

RASSF1

ENSG00000068028

-57 to +4 from start codon

CGCCCGGCCCGCGCTTGCTAGCGCCCAAAGCCAGCGAAGCACGGGCCCAACCGG
GCCATGTCGGGGGAGCCTGAGCTCATTGAGCTGCGGGAGCTGG

TP73

ENSG00000078900

+93 to 163 from TSS

AGGGGACGCAGCGAAACCGGGGCCCGCGCCAGGCCAGCCGGGACGGACGCCGAT
GCCCGGGGCTGCGACGGCTGCAGGTAGGAGGCCCAGGGCCGGGGGGCGGTTCGG
CTCCGCGGGCGGGGGCTGGAGCGCAGCGCTGGGCAGGCACCTGGGCTCGCAGCT
CCG
